# Supplementary material for: Printed smart devices for anti-counterfeiting allowing precise identification with household equipment
Source: Nat Commun. 2024 Feb 3;15:1040. doi: 10.1038/s41467-024-45428-3 (PMC10838302; doi:10.1038/s41467-024-45428-3)

Supplementary Materials

Printed smart devices for anti-counterfeiting allowing precise identification with household equipment

Junfang Zhang[1,2], *Rong Tan*[1,3], *Yuxin Liu*[1,4], *Matteo Albino*[2], *Weinan Zhang*[2], *Molly M. Stevens*[2], *and Felix F. Loeffler*[1]*

[1] Max Planck Institute of Colloids and Interfaces, Am Muehlenberg 1, 14476 Potsdam, Germany

[2] Department of Materials, Department of Bioengineering, and Institute of Biomedical Engineering, Imperial College London, London SW7 2AZ, United Kingdom

[3] Soochow University, College of Chemistry, Chemical Engineering and Material Science, 215123 Suzhou, China

[4] Freie Universität Berlin, Department of Chemistry and Biochemistry, Arnimallee 22, 14195 Berlin, Germany

*E-mail: Felix.Loeffler@mpikg.mpg.de

**Supplementary Information**	**Page**
**Figure S1** Process of generating a polymer spot pattern by laser-induced forward transfer	3
**Figure S2** Synthesis process of a PUF device	4
**Figure S3** Tuning the micro-hole pattern by the printing parameters during the generation of the polymer spot array	5
**Figure S4** Height map and profile of the thicker hematite film	6
**Figure S5** Resistance values of the electrodes obtained with different laser carbonization parameters	7
**Figure S6** PUF electronics synthesized by carbonization of styrene acrylic copolymer (s-LEC) under different laser scanning parameters	8
**Figure S7** PUF electronics synthesized by carbonization of PVP under different laser scanning parameters	9
**Figure S8** PUF electronics synthesized by carbonization of PVA under different laser scanning parameters	10
**Figure S9** PUF electronics synthesized with different carbonization repetitions	11
**Figure S10** Device uniqueness of the PUF patterns were characterized by inter-device Hamming distance	12
**Figure S11** Electrode resistance of the samples with different writing angles in reference to the direction of connection wires	13
**Figure S12** Characterization of device uniqueness, false authentication, and authentication error of the PUF patterns by LoFTR	14
**Figure S13** Quantitative analyses of the surface roughness (root-mean-square (RMS) height standard deviation) of the electrode areas before and after the treatments	15
**Figure S14** Semi-automatic resistance measurement setup	16
**Table S1** Comparison between the proposed PUF device and recently reported PUFs from the literature	17
**Table S2** PUF parameters for fluorescence and topography characterization	18
**Appendix:** 100 electronic devices for statistical analyses of PUF properties	19–22

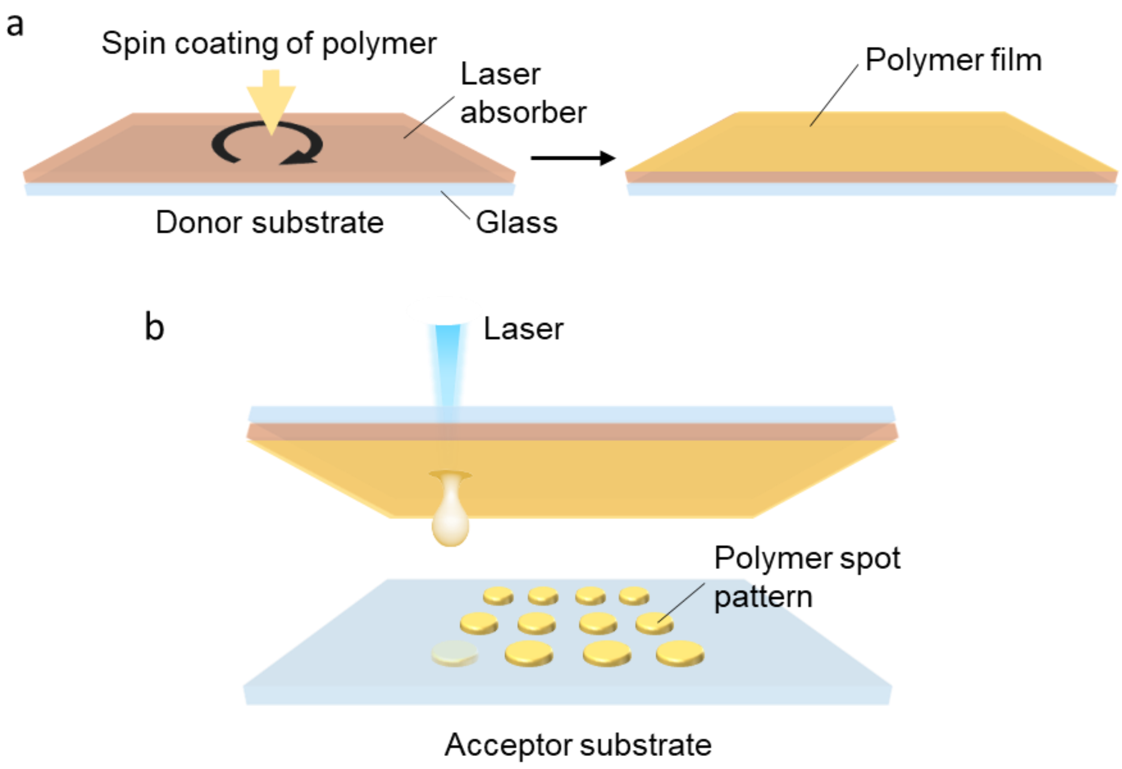

Figure S1. Process of generating a polymer spot pattern by laser-induced forward transfer (LIFT). (a) A donor glass substrate, covered with a (hematite) nanofilm laser absorber (~400 nm), is spin coated with a thin polymer (*e.g.*, polystyrene) film of ~200 nm. (b) The donor slide is placed onto a glass acceptor substrate (future PUF device) and a polymer spot pattern is transferred.

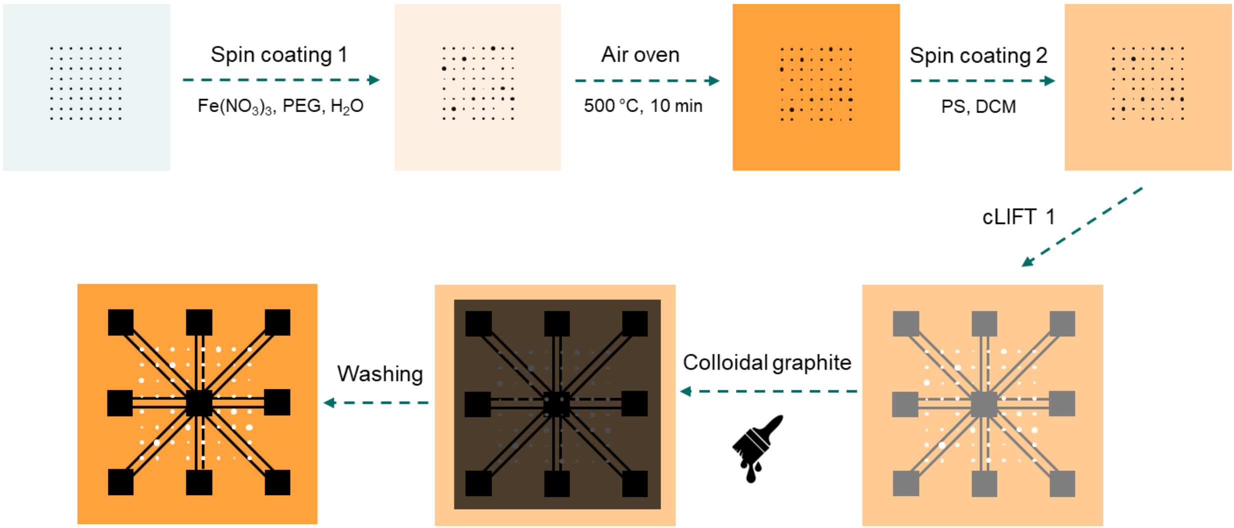

Figure S2. Synthesis process of a PUF device. A polymer spot array is generated on a glass substrate by LIFT. During the first spin-coating step, an iron nitrite solution randomly washes away some or parts of the polymer spots. After annealing at 500 °C for 10 min, a hematite nanofilm with micro-hole patterns is obtained. This (inhomogeneous) hematite layer functions as a laser absorber to carbonize the polymer films on top of it under laser irradiation. Then, the whole area is brushed with colloidal graphite, which is washed away together with the uncarbonized polymer.

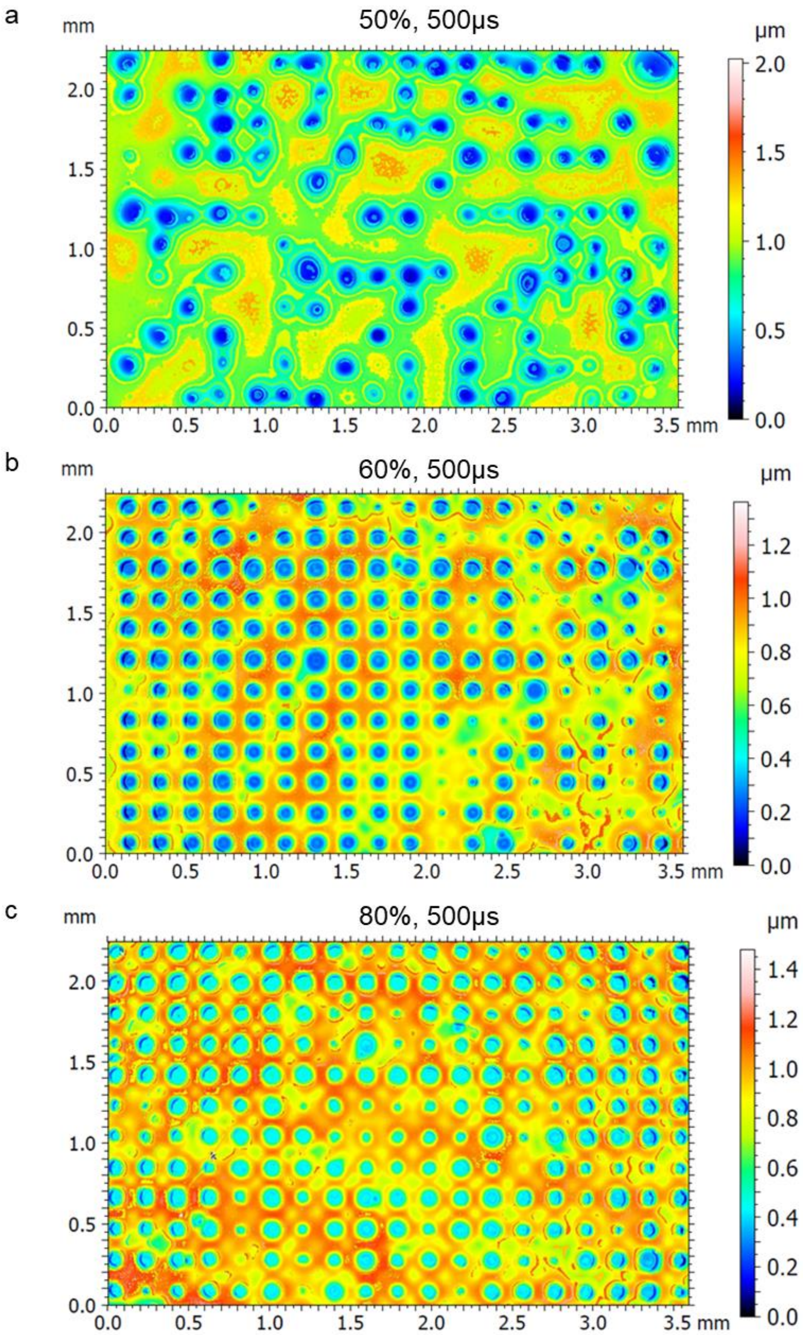

Figure S3. The micro-hole pattern can be tuned by the printing parameters during the generation of the polymer spot array. Spot patterns with (a) low (50 % = 55 mW), (b) medium (60 % = 63 mW), and (c) higher (80 % = 95 mW) laser power (all 500 µs irradiation per spot). Spots generated with lower laser power (a) tend to be washed away more easily during the first spin-coating step in contrast to those with higher laser power (b, c). Therefore, less micro-holes will be observed after annealing.

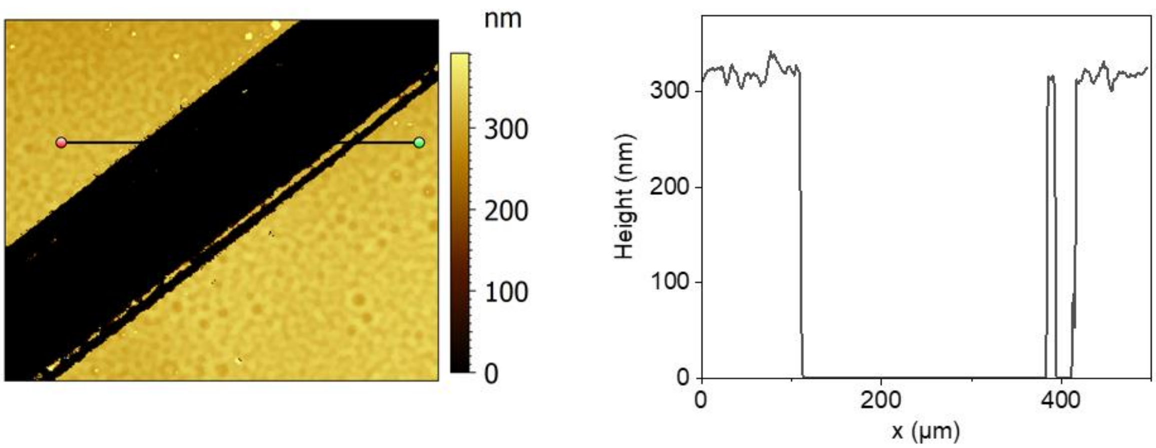

Figure S4. Height map and profile of the thicker hematite film.

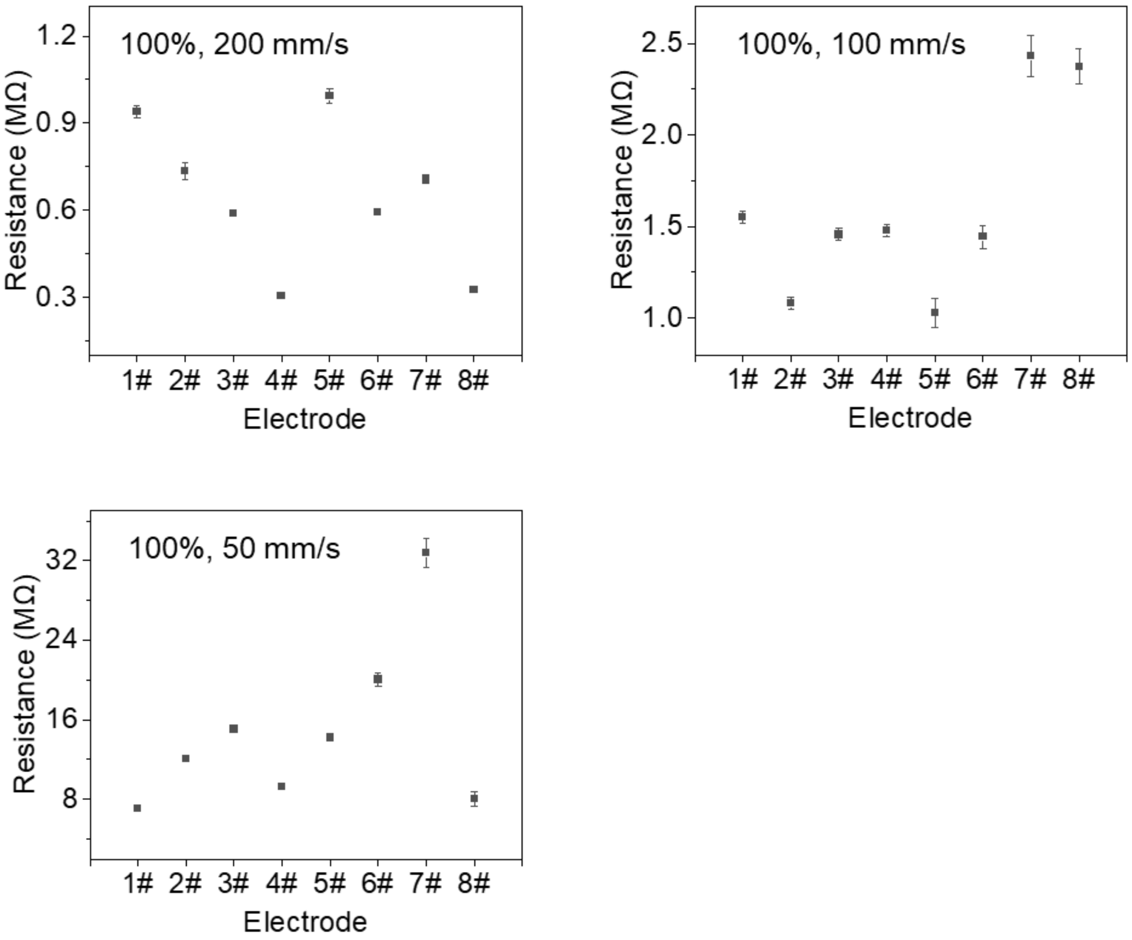

Figure S5. Resistance values of the electrodes obtained with different laser carbonization parameters (laser scanning speed 50, 100, 200 mm/s). Data shown as average of $n = 3$ measurements with standard deviation.

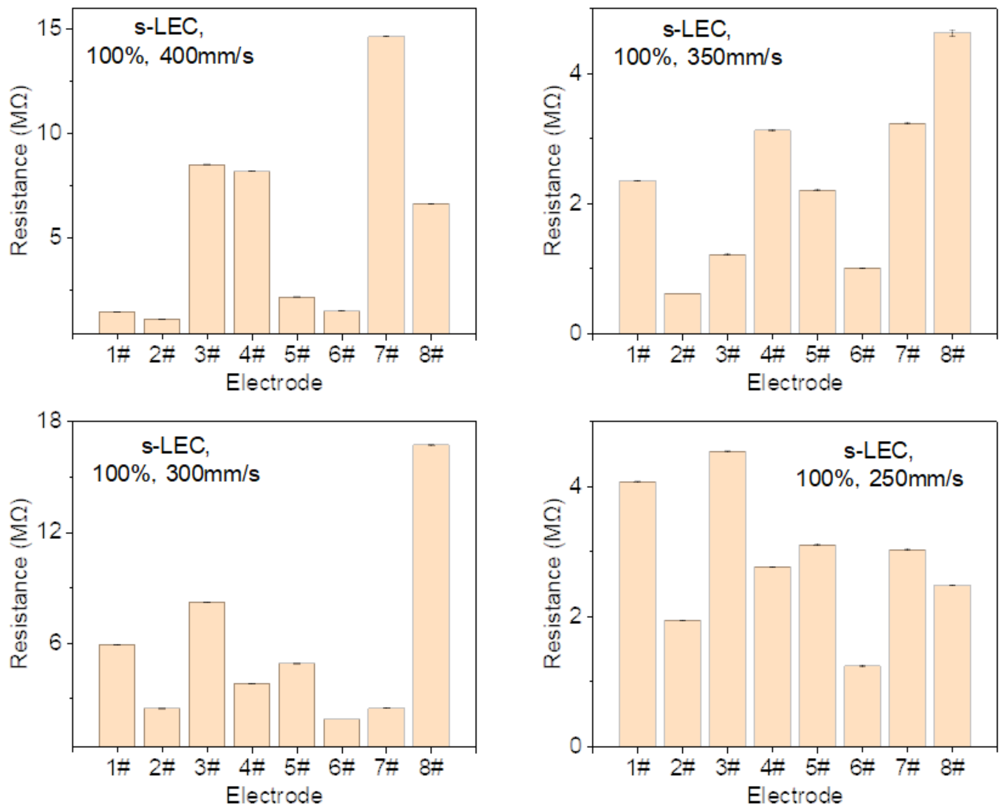

Figure S6. PUF electronics synthesized by carbonization of styrene acrylic copolymer (S-LEC) under different laser scanning parameters. Data shown as average of $n = 3$ measurements with standard deviation.

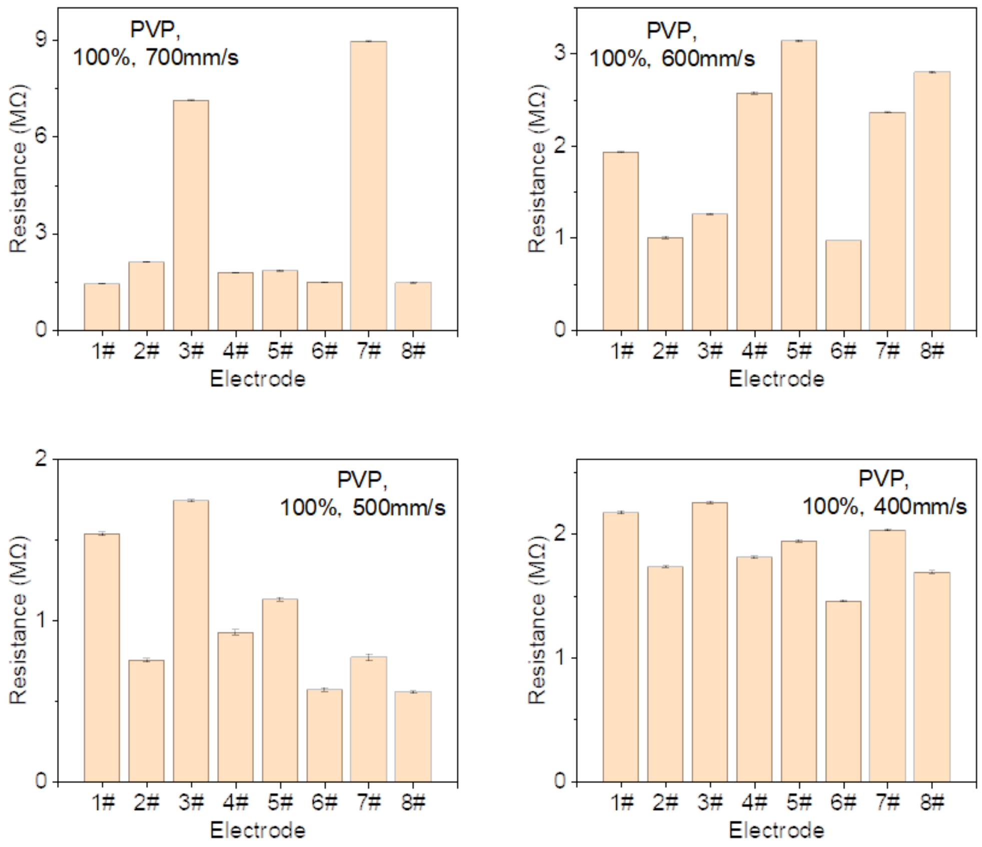

Figure S7. PUF electronics synthesized by carbonization of PVP under different laser scanning parameters. Data shown as average of $n = 3$ measurements with standard deviation.

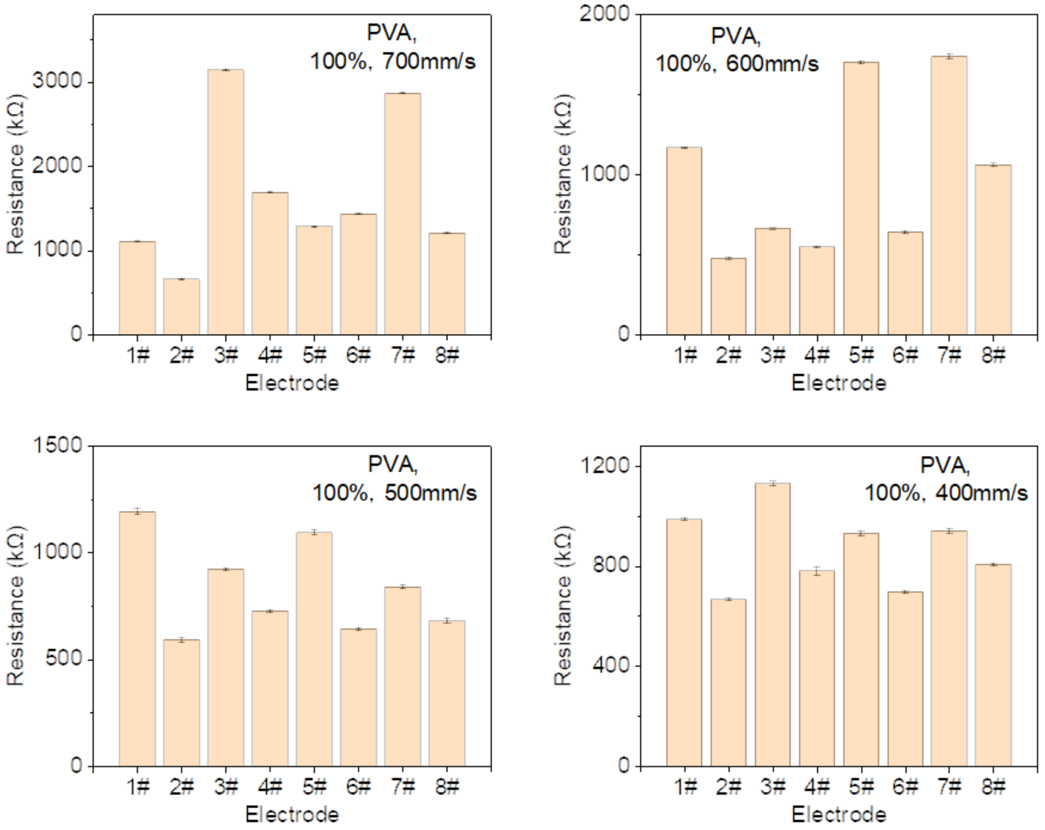

Figure S8. PUF electronics synthesized by carbonization of PVA under different laser scanning parameters. Data shown as average of $n = 3$ measurements with standard deviation.

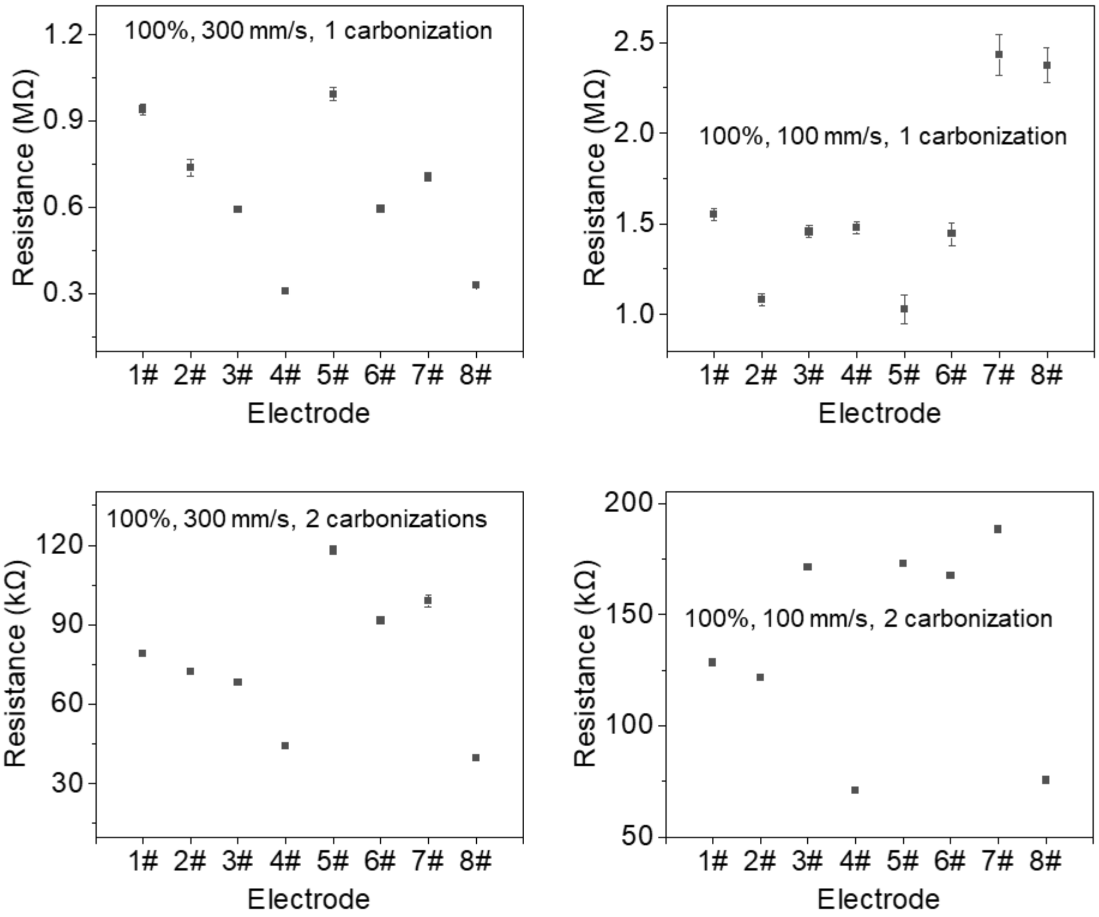

Figure S9. PUF electronics synthesized with different carbonization repetitions. When repeating the laser carbonization once (step 3 is only repeated for the electrode areas, not for the connection wires), the resistance tends to be decreased and more stable. Data shown as average of $n = 3$ measurements with standard deviation.

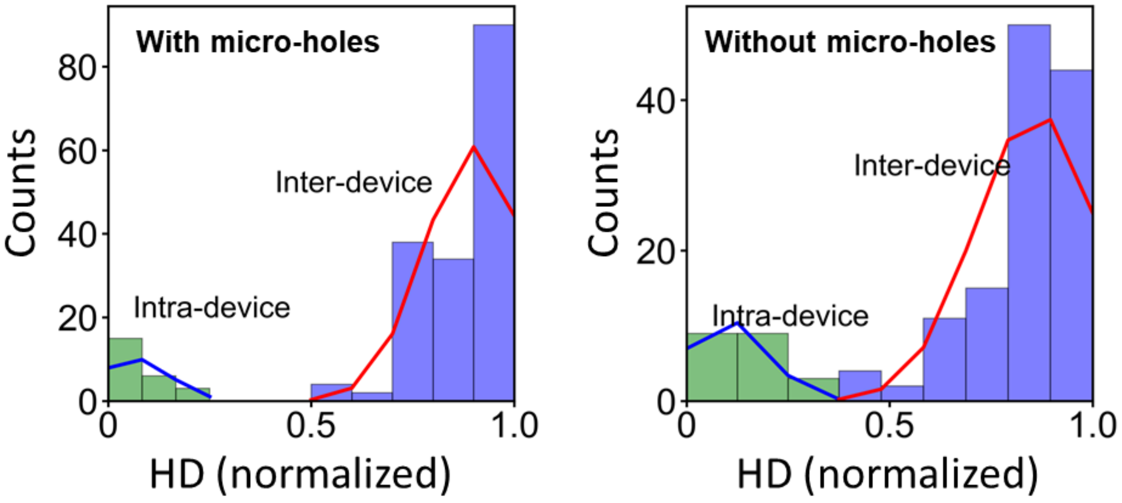

Figure S10. Device uniqueness of the PUF patterns were characterized by inter-device Hamming distance (HD). The readout reproducibility of the PUF patterns was characterized by the intra-device HD, where each PUF pattern was scanned three times.

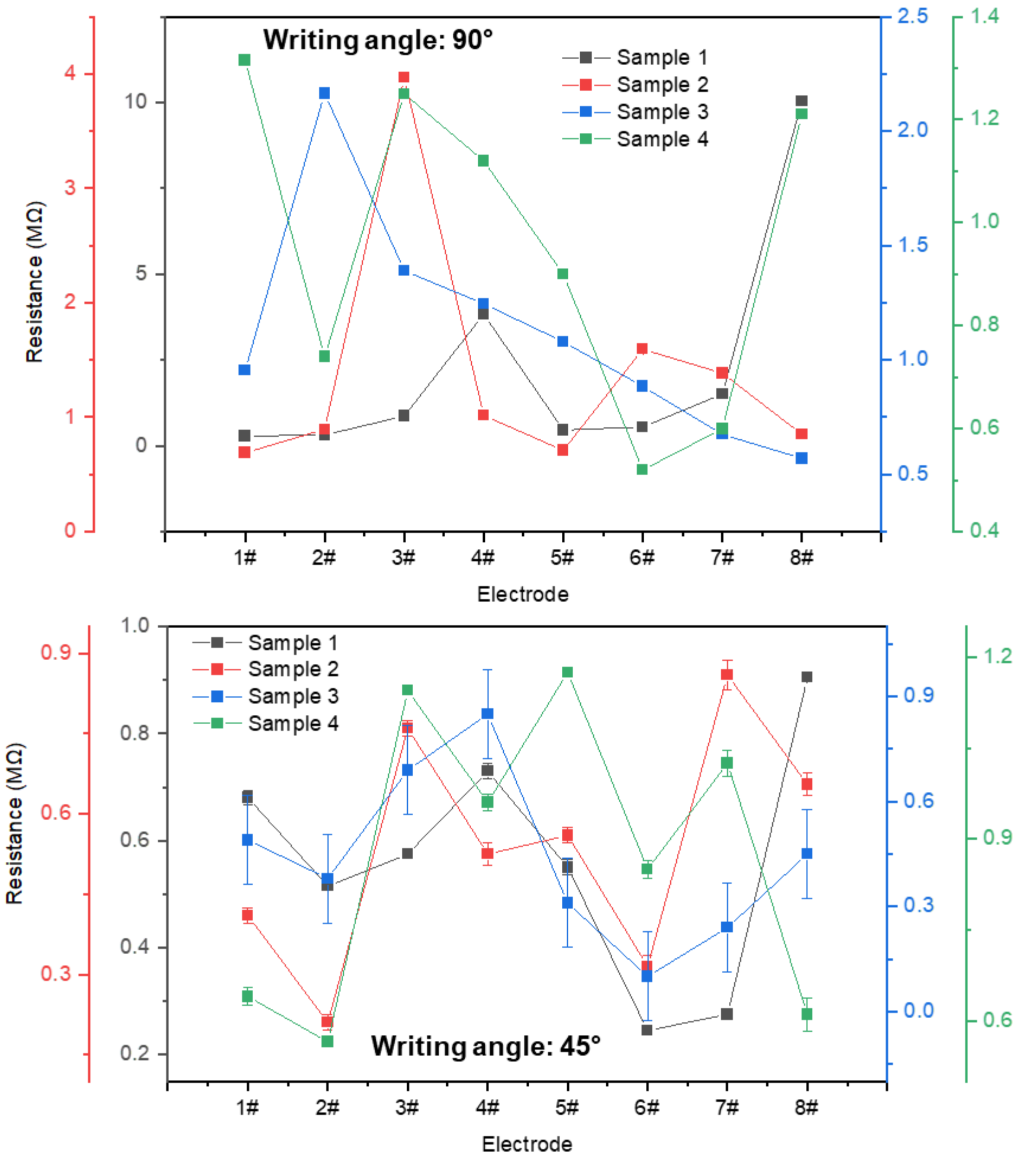

Figure S11. Electrode resistance (1# to 8#) of the samples with different writing angles in reference to the direction of connection wires. Each sample is measured three times. The mean value and standard error are shown in the figures.

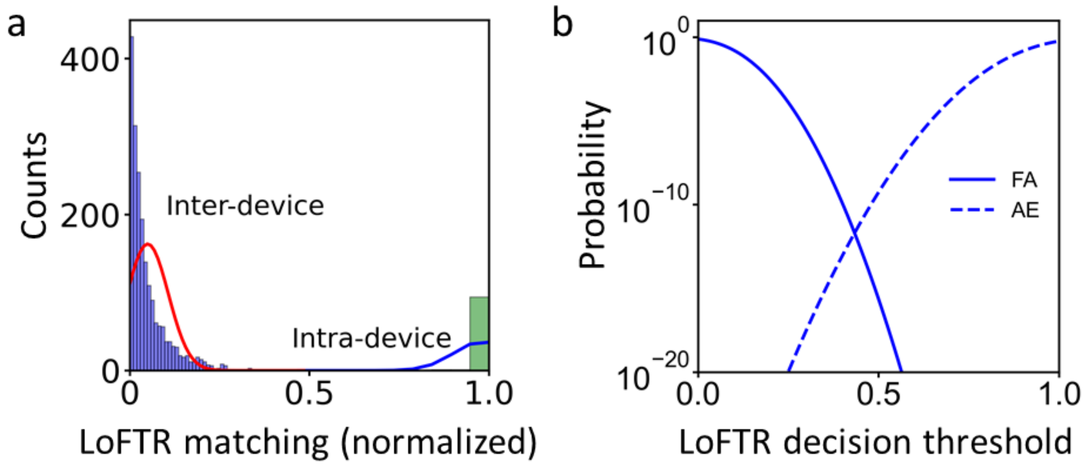

Figure S12. Characterization of device uniqueness, false authentication, and authentication error of the PUF patterns by LoFTR. (a) Device uniqueness of the micro-hole patterns were characterized by inter-device LoFTR similarity. The readout reproducibility of the micro-hole patterns was characterized by the intra-device LoFTR similarity, where each PUF pattern was scanned twice. (b) Cumulative distribution functions, showing the probabilities of false authentication (FA) and authentication error (AE) as a function of decision threshold. Details about LoFTR similarity calculations can be found in the Methods part of the manuscript.

	Before heat treatment	After heat treatment
**Electrode RMS roughness**	108.8 nm	103.6 nm
**Edge step height**	305.8 nm	283.5 nm
**VSI measurement area selection**	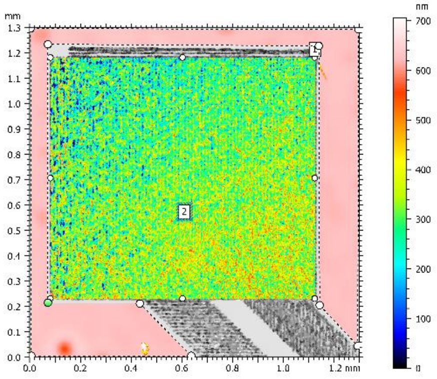	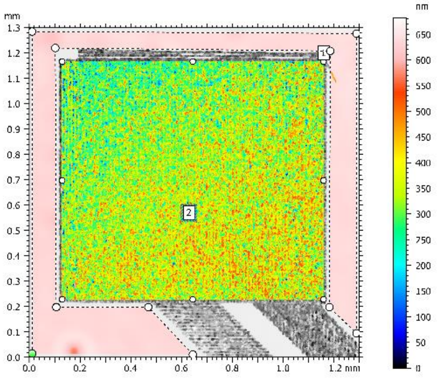

Before heat treatment:

Differential parameters	P2 - P1	Unit
Zmean(higher) - Zmean(lower)	305.8	nm
Angle difference	0.006433	°

Plane parameters	Unit	Plane 1	Plane 2
Zmin	nm	1.137e-13	108.9
ZMean	nm	319.5	675.3
Zmax	nm	705.7	705.7

After heat treatment:

Differential parameters	P2 - P1	Unit
Zmean(higher) - Zmean(lower)	283.5	nm
Angle difference	0.005592	°

Plane parameters	Unit	Plane 1	Plane 2
Zmin	nm	1.137e-13	216.0
ZMean	nm	351.5	635.0
Zmax	nm	687.0	652.5

	Before tape treatment	After tape treatment
**Electrode RMS roughness**	109.9 nm	108.7 nm
**Edge step height**	236.6 nm	271.2 nm
**VSI measurement area selection**	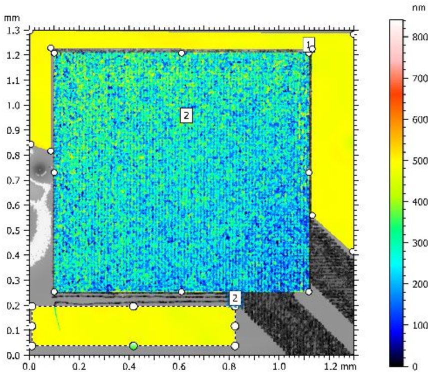	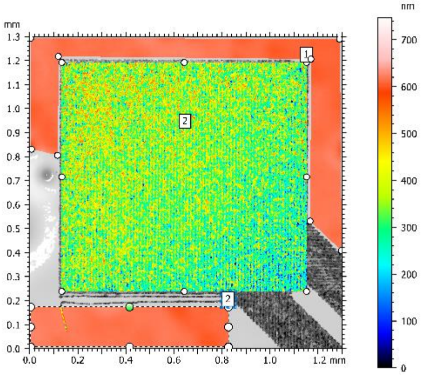

Before tape treatment:

Differential parameters	P2 - P1	Unit
Zmean(higher) - Zmean(lower)	236.6	nm
Angle difference	0.004462	°

Plane parameters	Unit	Plane 1	Plane 2
Zmin	nm	0.000	19.01
ZMean	nm	253.0	489.6
Zmax	nm	841.3	752.4

After tape treatment:

Differential parameters	P2 - P1	Unit
Zmean(higher) - Zmean(lower)	271.2	nm
Angle difference	0.005955	°

Plane parameters	Unit	Plane 1	Plane 2
Zmin	nm	0.000	199.4
ZMean	nm	342.7	613.9
Zmax	nm	746.5	636.6

Figure S13. Quantitative analyses of the surface roughness (root-mean-square (RMS) height standard deviation) of the electrode areas before and after the treatments. The RMS roughness differs only very slightly (<5 nm), while the edge step height differed a bit more (~20 – 35 nm) in the selected areas. This might be also influenced by the slight differences in the required manual selection of the measurement areas.

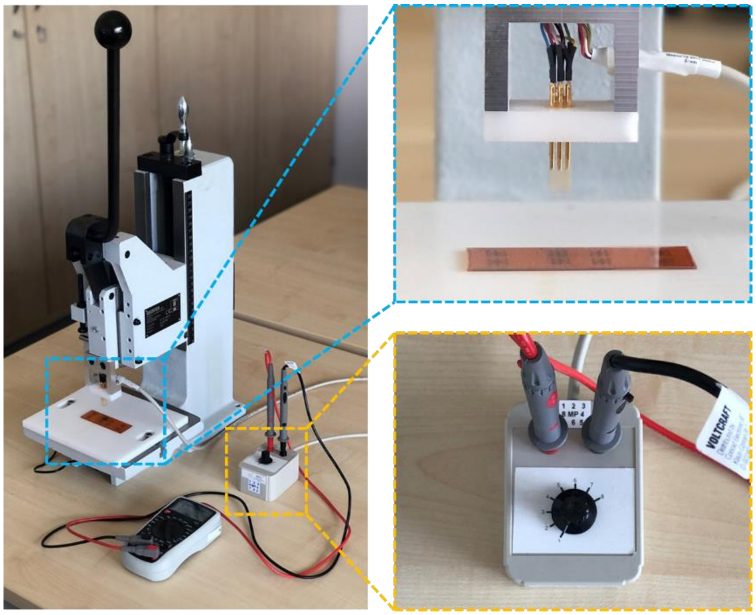

Figure S14. Semi-automatic resistance measurement setup. Nine metal pins are arranged in a 3 x 3 rectangle to fit the contact electrodes of our standard PUF devices. The pins are spring-loaded and a hand gear allows them to be moved up and down for quick contact. A window in the table, directly below the pins with an LED, allows for easy repositioning and alignment of the PUF on the table. A rotary selector switch allows manual selection of the circuit, which is connected to the ohmmeter.

Table S1. Comparison between the proposed PUF device and recently reported PUFs from the literature.

PUF Devices	Key generation	Equipment	Printing ability	String length	Deep learning-based identification	Reference
nanoPUF	optical dichroism; resistance; Raman	Semiconductor parameter analyzer; polarized optical microscope; dispersive Raman spectrometer	Non-printable	2	No	*Nat Electron* 5, 433–442 (2022)
fractalPUF	Optical pattern; Raman (conceptual presentation)	Optical microscope; Raman spectrometer	printable	2	Yes	*Nat Commun* 14, 2185 (2023)
Carbon dots PUF	Fluorescence pattern; thickness	Fluorescence microscope; white-light interferometry	printable	2	Yes	*Nat. Nanotechnol.* 18, 1027–1035 (2023)
laserPUF	Optical pattern	Optical microscope	printable	3	No	*Adv. Funct. Mater.* 33, 2211762 (2023)
**rPUF**	**Optical pattern, resistance**	**Ohmmeter; optical microscope**	**printable**	**16**	**Yes**	**This work**

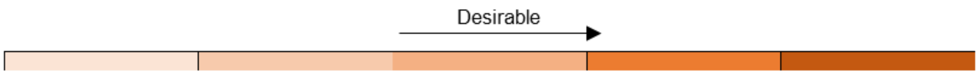

Table S2. PUF parameters for fluorescence and topography characterization. Calculated values are the average with standard deviation.

Method	Scan speed	Bit uniformity	Uniqueness	Reliability	Theoretical key space
**Optical microscope**	~s	0.481±0.056	0.454±0.057	0.805±0.024	$2^{416} \approx 2{\cdot}10^{125}$
**Ohmmeter**	~s	-	0.937±0.071	0.971±0.053	$16^{8} \approx 4{\cdot}10^{9}$
**Integrated**	~s	-	-	0.888±0.058	$\approx 7{\cdot}10^{134}$

Appendix: 100 electronic devices for statistical analyses of PUF properties

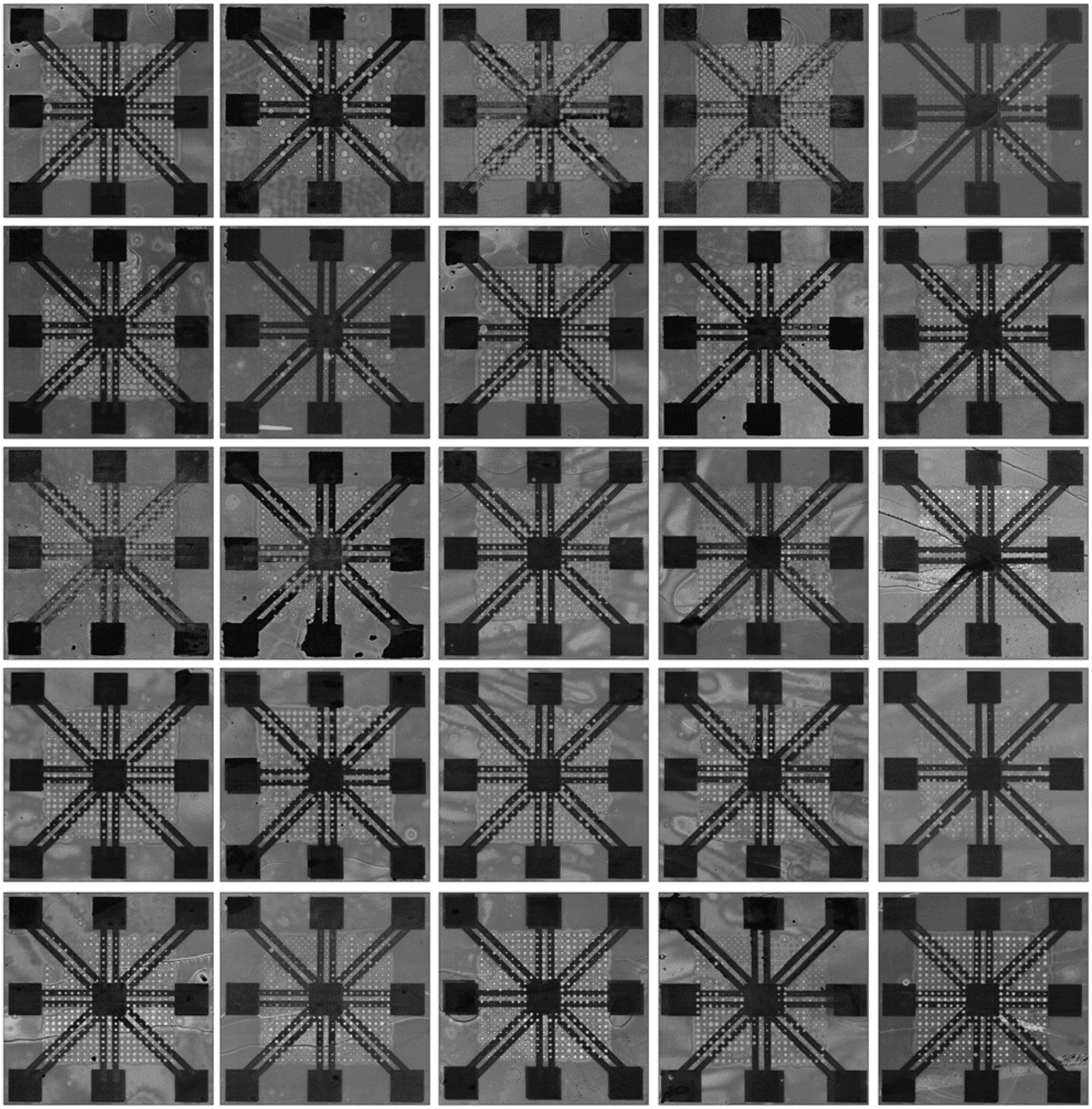

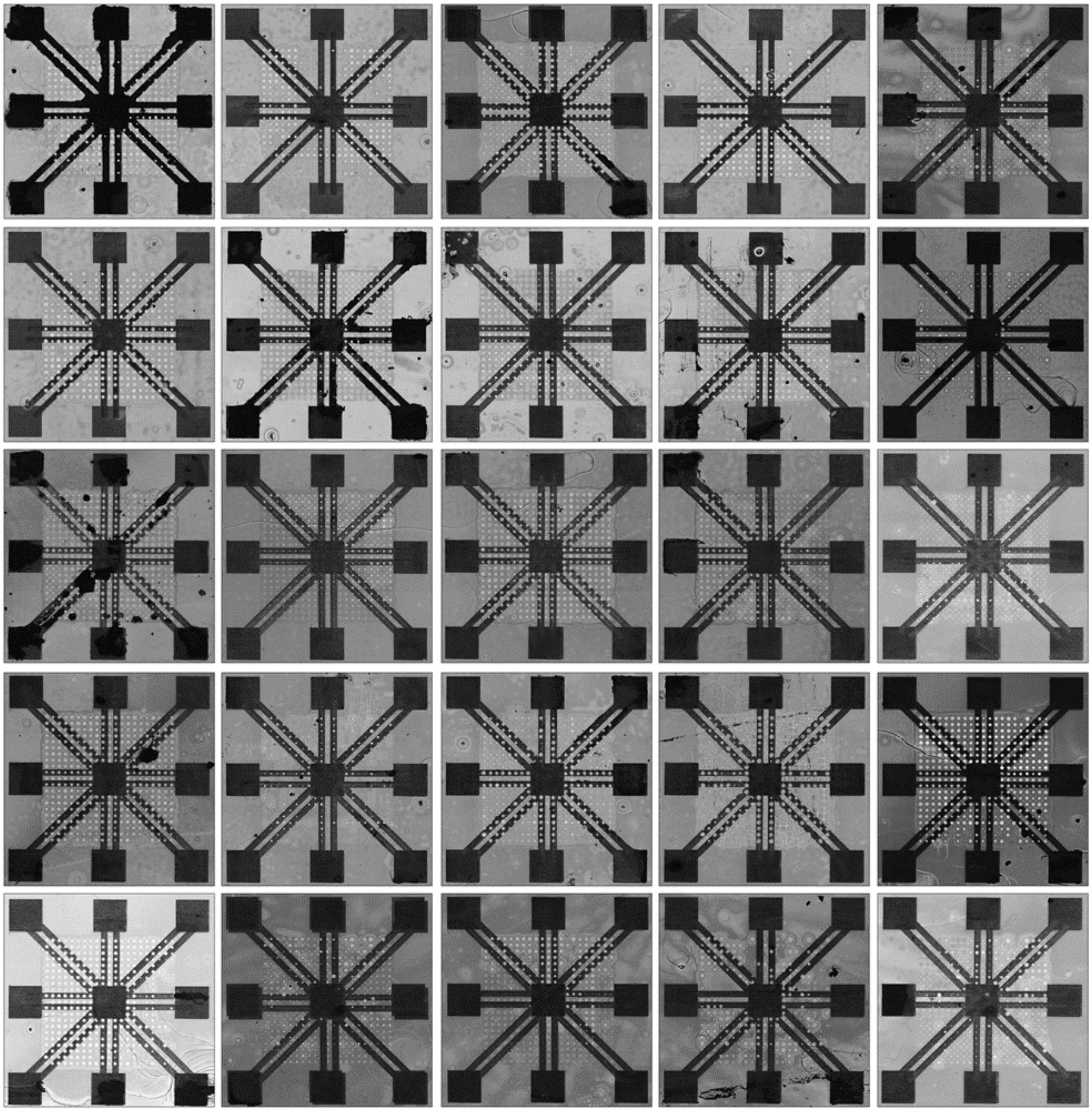

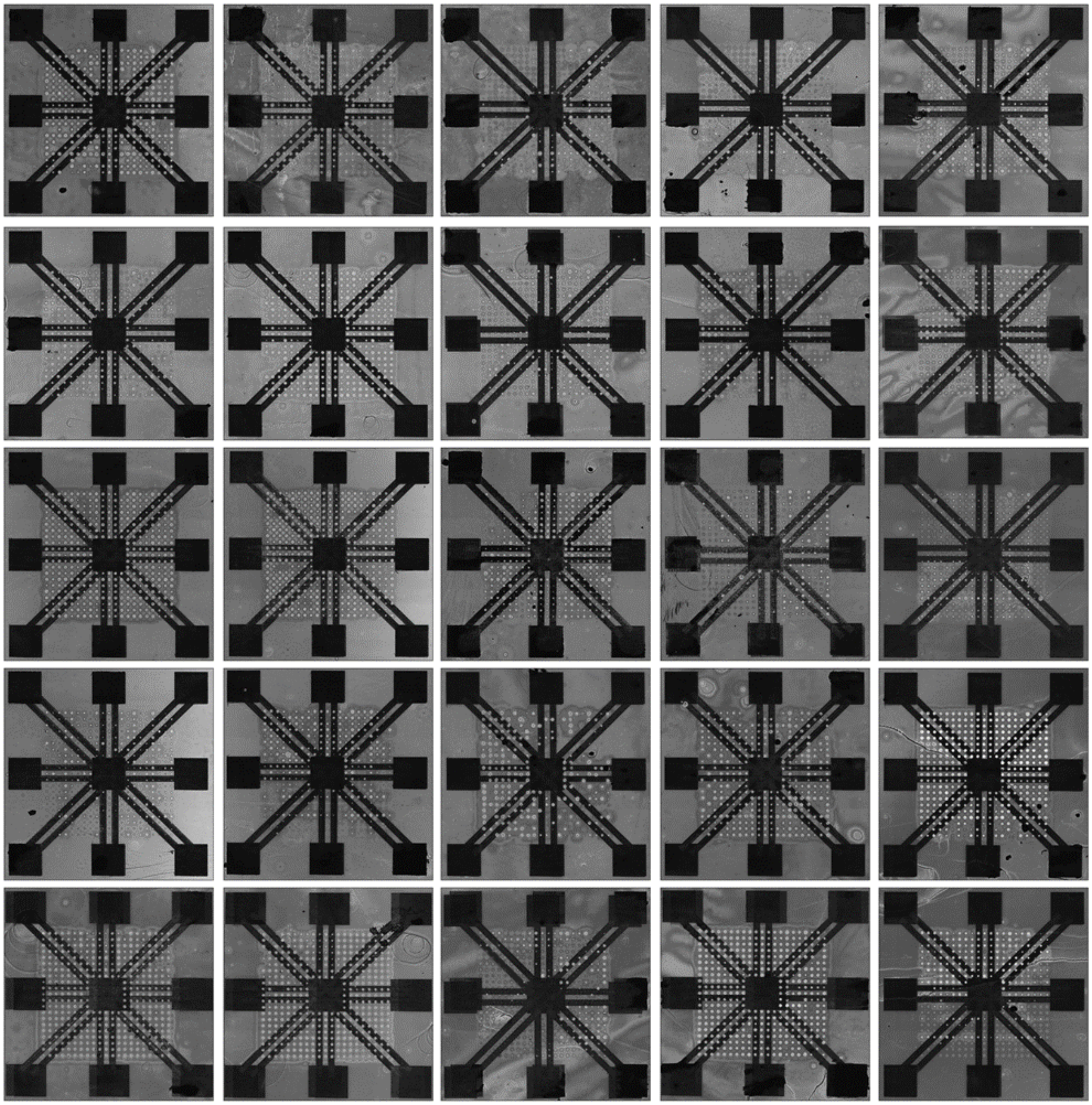

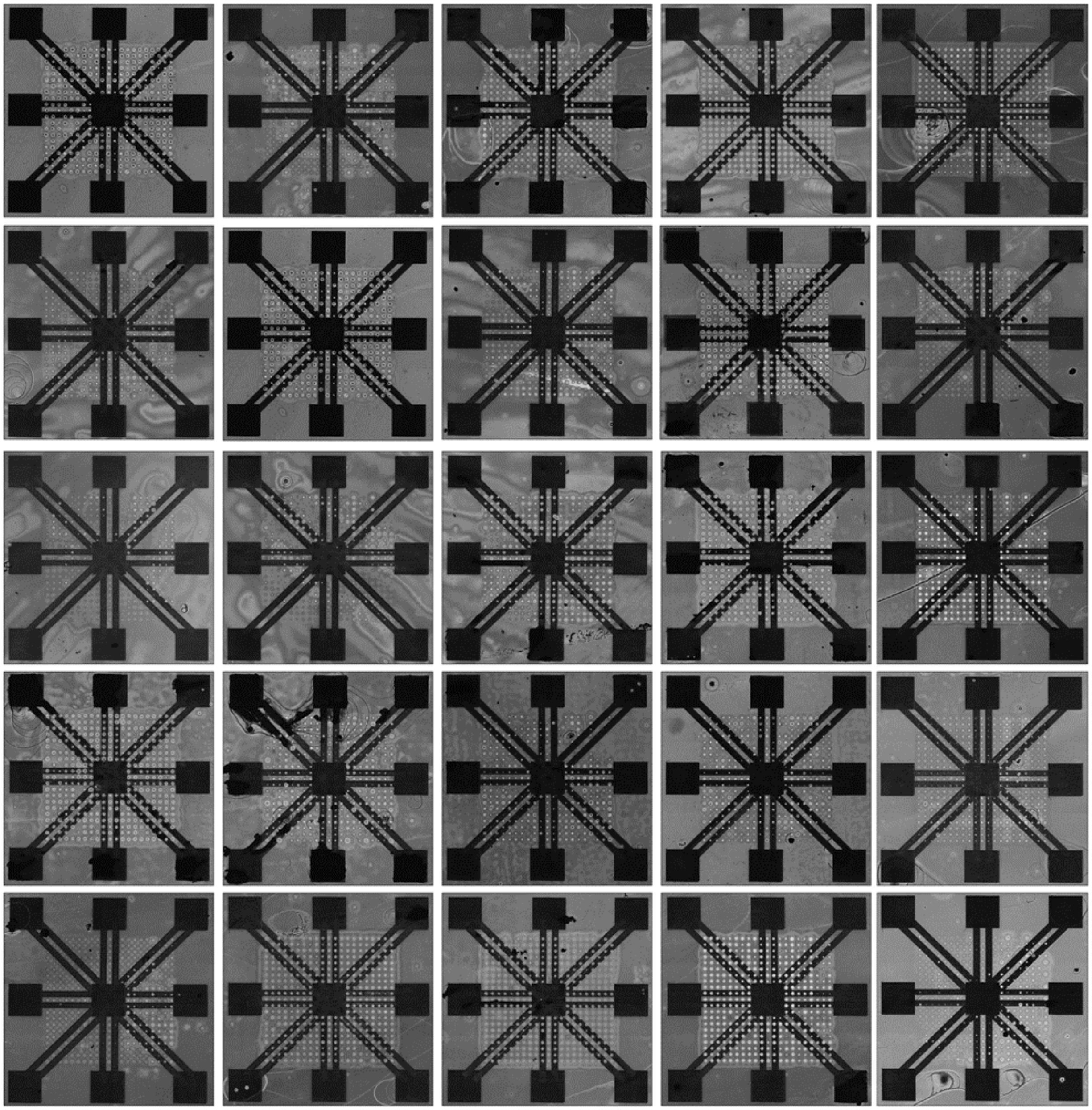

Supplement: Supplementary file 2 — Supplementary Information [file 41467_2024_45428_MOESM2_ESM.pdf]
